# Space-resolved chemical information from infrared extinction spectra

**DOI:** 10.1038/s41598-023-27619-y

**Published:** 2023-01-11

**Authors:** Yushmantha Ishan Kalpa de Silva Thuiya Hennadige, Proity Nayeeb Akbar, Reinhold Blümel

**Affiliations:** Present Address: Department of Physics, Wesleyan University, 265 Church Street, Middletown, CT 06459-0155 USA

**Keywords:** Infrared spectroscopy, Computational biophysics

## Abstract

A new method is presented for the extraction of the complex index of refraction from the extinction efficiency, $Q_{ext} {({\tilde{\nu }})}$, of homogeneous and layered dielectric spheres that simultaneously removes scattering effects and corrects measured extinction spectra for systematic experimental errors such as baseline shifts, tilts, curvature, and scaling. No reference spectrum is required and fit functions may be used that automatically satisfy the Kramers–Kronig relations. Thus, the method yields the complex refractive index of a sample for unambiguous interpretation of the chemical information of the sample. In the case of homogeneous spheres, the method also determines the radius of the sphere. In the case of layered spheres, the method determines the substances within each layer. Only a single-element detector is required. Using numerically computed $Q_{ext}({\tilde{\nu }})$ data of polymethyl-methacrylate and polystyrene homogeneous and layered spheres, we show that the new reconstruction algorithm is accurate and reliable. Reconstructing the complex refractive index from a published, experimentally measured raw absorbance spectrum shows that the new method simultaneously corrects spectra for scattering effects and, given shape information, corrects raw spectra for systematic errors that result in spectral distortions such as baseline shifts, tilts, curvature, and scaling.

## Introduction

With roots going back more than a century in time^1^, infrared (IR) spectroscopy has evolved into one of the most powerful and successful tools for the analysis of biological cells and tissues for applications in biophysics, chemistry, and medical pathology^2–6^. Far from being a successful, but stagnant field of study, with the recent advent of tunable IR laser sources^7^, IR spectroscopy is currently experiencing growing interest and expansion into new fields of application. No matter whether the spectra are taken with thermal, laser, or synchrotron IR sources, two tasks always have to be performed: (1) Cleaning up of the raw spectra and (2) reconstructing the refractive index (revealing the chemistry) of the (biological) sample under investigation. Powerful tools exist for cleaning up the spectra, i.e., removing artefacts introduced by the spectrographs and IR sources that result, e.g., in baseline shifts, tilts, curvature, and multiplicative distortions of the spectra^8^.

This paper focuses on single-cell IR spectroscopy where the cell sizes are approximately of the same order of magnitude as the wavelength of the incident IR radiation. Thus, the corresponding IR spectra exhibit pronounced scattering effects, such as Mie scattering, that modify absorbance spectra considerably and need to be removed before interpreting the spectra^8–12^.

In this paper we present a new technique that is capable of accomplishing the tasks (1) and (2) above without the need of a reference spectrum^8^ or the Kramers–Kronig relations^8,11^ as required by leading spectral correction methods. For our examples, we use two substances, polymethyl-methacrylate (PMMA) and polystyrene (PS).

## Methods

In “General” section we start by describing the general method used in IR spectroscopy. Then, in “Reconstruction of the complex refractive index” section, we describe a new method of reconstructing the space- and wavenumber-dependent (hyperspectral) complex index of refraction $\eta (\vec r,{\tilde{\nu }})$ based on anti-symmetrized Lorentzians. Here, $\vec r$ denotes the space part of the complex refractive index and ${{\tilde{\nu }}}=1/\lambda$ is the wavenumber, where $\lambda$ is the vacuum wavelength. In “Numerical simulations” section we describe our numerical procedures for reconstructing $\eta (\vec r,{{\tilde{\nu }}})$. In “The task at hand: an inverse scattering problem” section we explain how simple curve-fitting methods differ from reconstruction methods, which belong to the class of inverse-scattering problems. In “Quality of reconstructions” section we define our method for assessing the quality of reconstruction.

### General

Typically in single-detector IR spectroscopy, a beam of IR radiation with wavenumber ${{\tilde{\nu }}}$ and intensity $I_0({{\tilde{\nu }}})$ is directed toward a target that may be a biological cell, a sample of biological tissue, or a grain of biological or inanimate matter, and the transmitted intensity $I({{\tilde{\nu }}})$ is measured in forward direction. Because of absorption of IR radiation by the target, and because of scattering of IR radiation out of the forward direction, we have $I({{\tilde{\nu }}}) <I_0({{\tilde{\nu }}})$. Once $I({{\tilde{\nu }}})$ is measured, the result of the measurements is typically reported as the apparent absorbance

1 $$\begin{aligned} {{\mathscr {A}}}({{\tilde{\nu }}}) = -\log _{10} \left[ \frac{I({{\tilde{\nu }}})}{I_0({{\tilde{\nu }}})} \right] . \end{aligned}$$

While the apparent absorbance ${{\mathscr {A}}}$ is convenient for recording and reporting experimental results, cross sections are more convenient for the analysis of experiments, numerical simulations, and the extraction of refractive indexes. Denoting by $\sigma _{\text{scat}}$ and $\sigma _{\text{abs}}$ the scattering and absorption cross sections, respectively, the extinction cross section is defined as^13^ $\sigma _{\text{ext}} = \sigma _{\text{scat}} + \sigma _{\text{abs}}$. With $\sigma _{\text{ext}}$, we define the extinction efficiency according to Ref.^13^ $Q_{\text{ext}} = \sigma _{\text{ext}}/g$, where *g* is the geometric cross section of the sample under investigation. Denoting by $G\gg g$ the reception area of the detector, the radiative power received by the detector without the sample present is $P_0 = G I_0$, and the radiative power received by the detector with the sample present is $P_I=GI= GI_0-I_0\sigma _{ext}$. Rearranging this equation and dividing by *g*, we obtain

2 $$\begin{aligned} Q_{\text{ext}}({{\tilde{\nu }}}) = \frac{\sigma _{\text{ext}}({{\tilde{\nu }}})}{g} = \frac{GI_0({{\tilde{\nu }}})- GI({{\tilde{\nu }}})}{gI_0({{\tilde{\nu }}})} =\left( \frac{G}{g}\right) \left[ 1-\left( \frac{I({{\tilde{\nu }}})}{I_0({{\tilde{\nu }}})} \right) \right] . \end{aligned}$$

Via (1), this can immediately be related to the apparent absorbance ${{\mathscr {A}}}$ according to

3 $$\begin{aligned} Q_{\text{ext}}({{\tilde{\nu }}}) = \left( \frac{G}{g}\right) \left[ 1-10^{-{{\mathscr {A}}({{\tilde{\nu }}})}} \right] . \end{aligned}$$

Since, via (3), $Q_{\text{ext}}({{\tilde{\nu }}})$ and ${{\mathscr {A}}}({{\tilde{\nu }}})$ contain the same information, we will, in the following, present our theoretical analysis and reconstructions of refractive indexes on the basis of $Q_{\text{ext}}({{\tilde{\nu }}})$ or ${{\mathscr {A}}}({{\tilde{\nu }}})$ depending on convenience.

**Figure 1:**
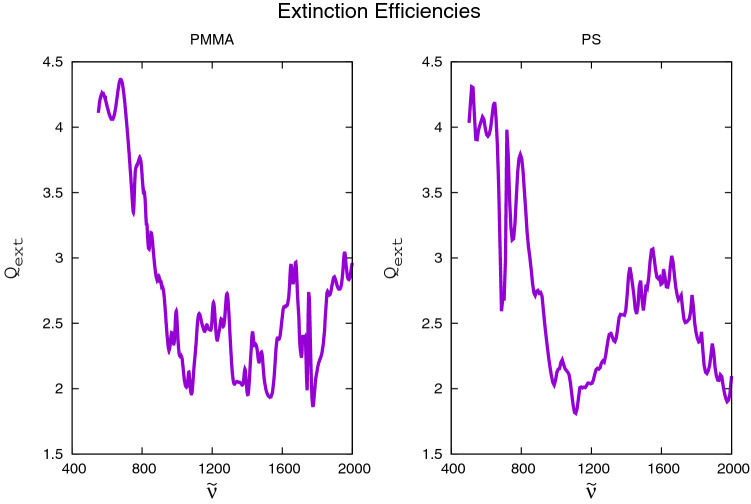
Extinction efficiency $Q_{\text{ext}}({{\tilde{\nu }}})$, numerically computed^16^ on the basis of published refractive-index data^14,15^ for a sphere of radius $10\,\upmu$m, homogeneously filled with PMMA (left panel) and PS (right panel).

As an example, we show in Fig. 1 the numerically computed $Q_{\text{ext}}({{\tilde{\nu }}})$ spectrum for the experimentally determined PMMA refractive-index data published in Ref.^14^ (left panel) and the $Q_{\text{ext}}({{\tilde{\nu }}})$ spectrum for the experimentally determined PS refractive-index data published in Ref.^15^ (right panel). The computations of $Q_{\text{ext}}({{\tilde{\nu }}})$ in Fig. 1 were performed with a standard Mie code^16^.

**Figure 2:**
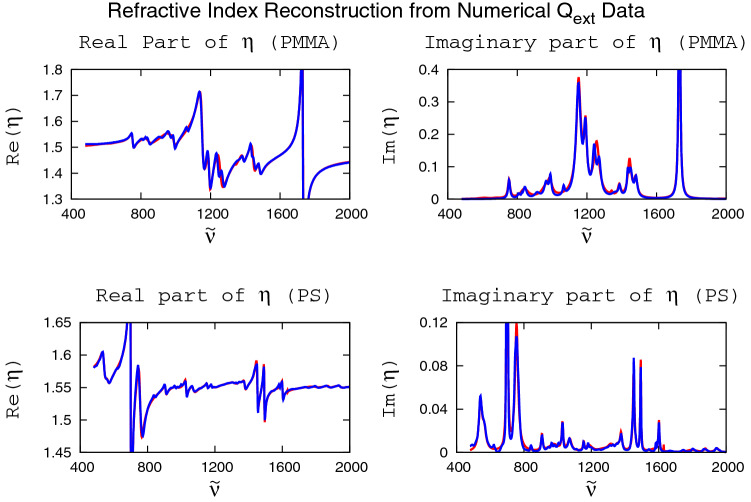
Reconstructions of real parts (left panels) and imaginary parts (right panels) of the complex refractive index $\eta ({{\tilde{\nu }}})$ of $10\,{\upmu }$m radius spheres, homogeneously filled with PMMA (upper panels) and PS (lower panels), using experimental data (blue lines) for obtaining $Q_{\text{ext}}^{(\text{given})} ({{\tilde{\nu }}})$. 18 Lorentzians were used for the PMMA reconstructions and 22 Lorentzians were used for the PS reconstructions. Although the initial guess of the complex refractive indexes is random, the reconstructions (red lines) are near perfect, indicated by the near perfect overlap of the blue and red lines.

We see that the $Q_{\text{ext}}$ curves in Fig. 1 have very little in common with their respective refractive indexes (see blue lines in Fig. 2). In particular, the prominent peaks at small wavenumbers in both $Q_{\text{ext}}({{\tilde{\nu }}})$ curves in Fig. 1 are completely absent in the refractive-index data shown as the blue lines in Fig. 2. These peaks in $Q_{\text{ext}}({{\tilde{\nu }}})$ are caused by scattering and obscure, to a large extent, the underlying refractive index. The same holds, e.g., for the large undulatory structure in the PS $Q_{\text{ext}}({{\tilde{\nu }}})$ curve in Fig. 1 (right panel) in the vicinity of $1600\,\text{cm}^{-1}$, which does not have a counterpart in the refractive-index data (blue curves in Fig. 2). Thus, the challenge for any reconstruction algorithm is to recover both the real and imaginary parts of the refractive index (two curves) from the single, real input curve $Q_{\text{ext}}({{\tilde{\nu }}})$.

In any reconstruction problem, what is given, and it is the only input given, is the measured or computed extinction efficiency $Q_{\text{ext}}({{\tilde{\nu }}})$, as shown, for example, in Fig. 1. Thus, we denote such a given extinction efficiency by $Q_{\text{ext}}^{(\mathrm given)}({{\tilde{\nu }}})$. $Q_{\text{ext}}^{(\mathrm given)}({{\tilde{\nu }}})$ is the starting point of any reconstruction algorithm.

### Reconstruction of the complex refractive index

The main point of the new reconstruction method is to extract the space-dependent complex index of refraction $\eta (\vec r,{{\tilde{\nu }}})$ from $Q_{\text{ext}}({{\tilde{\nu }}})$. This may sound ambitious, since $Q_{\text{ext}}({{\tilde{\nu }}})$ has no space dependence. Nevertheless, it is possible, at least for a sphere with two layers, as demonstrated in “Layered sphere: space-resolved refractive index” section. To implement the method, we discretize space into voxels, which can be any shape or size. The smaller the voxel, the more resolution. The more regular the voxel (such as cubes or shells), the more straightforward the computations. Labeling the voxels with the discrete index $j=1,\ldots ,J$, and denoting by $\vec r_j$ a representative point of voxel number *j* (for instance its center in the case of cubes or a point on the center shell in the case of shells), we represent the refractive index inside of a voxel by its (spatially) constant value $\eta (\vec r_j,{{\tilde{\nu }}}) \equiv \eta _j({{\tilde{\nu }}})$, and assume that its imaginary part varies in frequency as a sum of anti-symmetrized Lorentzians of the form^17^

4 $$\begin{aligned} \Im \left[ \eta _j({{\tilde{\nu }}}) \right] = \sum _{m=1}^M \left\{ \frac{h_m^{(j)}}{1+\bigg [({{\tilde{\nu }}}- {{\tilde{\nu }}}_m^{(j)})/ \Gamma _m^{(j)}\bigg ]^2} - \frac{h_m^{(j)}}{1+\bigg [({{\tilde{\nu }}}+ {{\tilde{\nu }}}_m^{(j)})/ \Gamma _m^{(j)}\bigg ]^2} \right\} , \ \ \ j=1,\ldots ,J, \ \end{aligned}$$

where the symbol $\Im$ denotes the imaginary part. Inside voxel number *j*, ${{\tilde{\nu }}}_m^{(j)}$ is the wavenumber location of an IR absorption band, $h_m^{(j)}$ is the corresponding peak height, $\Gamma _m^{(j)}$ is the width of the absorption band, $m=1,\ldots ,M$ numbers the absorption bands, and *M*, a parameter, decides how many bands we include in our reconstructions. ${{\tilde{\nu }}}_m^{(j)}$, $h_m^{(j)}$, and $\Gamma _m^{(j)}$, $m=1,\ldots ,M$, $j=1,\ldots ,J$, are adjustable parameters to be determined via a 3D scattering code such that $Q_{\text{ext}}^{(\text{model})}({{\tilde{\nu }}})$, i.e. the $Q_{\text{ext}}({{\tilde{\nu }}})$ predicted on the basis of (4), optimally reproduces $Q_{\text{ext}}^{(\text{given})} ({{\tilde{\nu }}})$, where $Q_{\text{ext}}^{(\text{given})} ({{\tilde{\nu }}})$ is the given $Q_{\text{ext}}({{\tilde{\nu }}})$, either obtained experimentally, or from a numerical simulation (see “Results” section). As pointed out by Keefe^17^, the anti-symmetrization in (4) is necessary so that the refractive index $\eta (\vec r,{{\tilde{\nu }}})$ satisfies the Kramers–Kronig conditions. Satisfying the Kramers–Kronig conditions is necessary because of causality requirements^17^. Consequently, the imaginary part of $\eta (\vec r,{{\tilde{\nu }}})$ needs to be anti-symmetric with respect to ${{\tilde{\nu }}}\rightarrow -{{\tilde{\nu }}}$, i.e., $\Im \eta (\vec r,-{{\tilde{\nu }}})= -\Im \eta (\vec r,{{\tilde{\nu }}})$. This is guaranteed by the form of (4).

The real part of $\eta (\vec r,{{\tilde{\nu }}})$ is obtained by the Kramers-Kronig transformation of (4), i.e.^17^,

5 $$\begin{aligned} \Re [\eta_j ({{\tilde{\nu }}})] - n_{\infty }&= \frac{1}{\pi } \int _{-\infty }^{\infty } \frac{\Im [\eta_j ({{\tilde{\nu }}}')]}{{{\tilde{\nu }}}'-{{\tilde{\nu }}}}\, d{{\tilde{\nu }}}' \nonumber \\&=-\sum _{m=1}^M \left\{ \frac{h_m^{(j)}({{\tilde{\nu }}} - {{\tilde{\nu }}}_m^{(j)})/\Gamma }{1+[({{\tilde{\nu }}}- {{\tilde{\nu }}}_m^{(j)})/ \Gamma _m^{(j)}]^2} - \frac{h_m^{(j)}({{\tilde{\nu }}} + {{\tilde{\nu }}}_m^{(j)})/\Gamma }{1+[({{\tilde{\nu }}}+ {{\tilde{\nu }}}_m^{(j)})/ \Gamma _m^{(j)}]^2} \right\} , \ \ \ j=1,\ldots ,J, \ \end{aligned}$$

where the symbol $\Re$ denotes the real part and $n_{\infty }$ is a free (real) parameter. It denotes the average real refractive index that originates from spectral domains, such as the optical frequency domain, which are outside the IR domain. The important point here is that the Kramers–Kronig transform of the anti-symmetrized Lorentzians is known analytically [see (5)] and does not have to be computed numerically. This is significant, since in most mid-IR experiments data are only taken in the spectral range from about ${{\tilde{\nu }}}\approx 500\,\text{cm}^{-1}$ to ${{\tilde{\nu }}}\approx 4000\,\text{cm}^{-1}$. This is insufficient for computing a high-accuracy Kramers–Kronig transform, which, according to (5), requires integration over an infinite range of wavenumbers.

In the simplest case, the entire scatterer may be represented as a single voxel, in which case $J=1$. This is appropriate for homogeneous scatterers, i.e., scatterers with a spatially constant index of refraction, such as a homogeneous sphere. An example with $J=2$ is a sphere with two layers, i.e., a core and a shell, each homogeneously filled with a medium of spatially constant index of refraction as discussed in “Results” section. In this case two voxels, i.e., the core and the shell, suffice, each endowed with its own series (4) and (5) of Lorentzians. The method now determines the unknown parameters ${{\tilde{\nu }}}_m^{(j)}$, $h_m^{(j)}$, and $\Gamma _m^{(j)}$, $m=1,\ldots ,M$, $j=1,\ldots ,J$, as the optimum values that best reproduce $Q_{\text{ext}} ^{(\text{given})} ({{\tilde{\nu }}})$. This can be done with any standard nonlinear fit algorithm that minimizes a target function *S*, for instance (the $\{\ldots \}$ notation indicates the entire set of fit parameters)

6 $$\begin{aligned} S\bigg [\{{{\tilde{\nu }}}_m^{(j)}, h_m^{(j)}, \Gamma _m^{(j)}\},\{p\}\bigg ] = \sum _{l} \left\{ Q_{\text{ext}} ^{(\text{model})} \bigg [\{{{\tilde{\nu }}}_m^{(j)}, h_m^{(j)}, \Gamma _m^{(j)}\},\{p\}; {{\tilde{\nu }}}_l\bigg ] - Q_{\text{ext}} ^{(\text{given})} ({{\tilde{\nu }}}_l) \right\} ^2, \end{aligned}$$

where $Q_{\text{ext}} ^{(\text{model})} [\{{{\tilde{\nu }}}_m^{(j)}, h_m^{(j)}, \Gamma _m^{(j)}\}, \{p\};{{\tilde{\nu }}}_l]$ is the extinction efficiency evaluated at ${{\tilde{\nu }}}_l$ with fit parameters ${{\tilde{\nu }}}_m^{(j)}$, $h_m^{(j)}$, and $\Gamma _m^{(j)}$, $m=1,\ldots ,M$, $j=1,\ldots ,J$, computed via a suitable 3D scattering code, and ${{\tilde{\nu }}}_l$ are the wavenumbers at which $Q_{\text{ext}} ^{(\text{given})}({{\tilde{\nu }}})$ has been measured or pre-determined numerically. The set $\{p\}$ is an additional set of parameters that always includes $n_{\infty }$ and may include additional fit parameters describing experimental spectral distortions such as baseline shifts, tilts, curvature, and scaling. These parameters can then be used to unfold the experimental extinction efficiency, i.e., automatically correct the experimental extinction spectrum simultaneously with extracting the complex refractive index, as done, e.g., in “Reconstruction from experimental extinction data” section for experimental spectral distortions.

The main characteristic of the Lorentzian method is that, in contrast to other spectral-correction methods, no reference spectrum is needed to scatter-correct $Q_{\text{ext}}({{\tilde{\nu }}})$ spectra, whether simulated via a forward model or obtained experimentally, and that no numerical Kramers–Kronig transformation is needed. This is so, since, according to (5), the Kramers–Kronig transform of the Lorentzian functions in (4) is known exactly and analytically.

### Numerical simulations

To generate the reconstructions whose results are reported in “Results” section, we implemented the algorithm described in “Reconstruction of the complex refractive index” section, using the MATLAB nonlinear least-squares fit routine lsqcurvefit. One characteristic feature of our implementation of the Lorentzian method is that, initially, as starting conditions, we place *M* Lorentzians, equi-spaced (unbiased) into the wavenumber interval under consideration. This way, in addition to using random initial values for Lorentzian peak heights and peak widths, we obtain an initial condition that is not only unbiased, but an initial Lorentzian peak is always close to an actual peak in the imaginary part of the refractive index, which then needs only a relatively small adjustment in position, height, and width, to morph into an actual peak. This feature is crucial. It avoids, to a large extent, getting trapped in secondary optimization minima, i.e., a local but not global minimum of the target function *S* [see (6)]. We can still get trapped in secondary minima, but because of our choice of equi-spaced initial Lorentzian positions, the chance of getting trapped in a secondary minimum that results in a bad fit is vastly reduced. As a consequence, we found that the algorithm is insensitive to the choice of initial conditions and yields excellent results even if initial heights and widths are chosen randomly.

### The task at hand: an inverse scattering problem

It may seem that the problem of recovering the refractive index from the extinction efficiency $Q_{\text{ext}}({{\tilde{\nu }}})$ is a simple problem of fitting Lorentzians to a given refractive index $\eta (\vec r,{{\tilde{\nu }}})$. This is not so. For a given (biological) sample, it is not the refractive index that is given or measured, it is the extinction efficiency $Q_{\text{ext}}({{\tilde{\nu }}})$ that is measured, while the refractive index $\eta (\vec r,{{\tilde{\nu }}})$ is unknown and needs to be found. The task, therefore, is to uncover the unknown refractive index $\eta (\vec r,{{\tilde{\nu }}})$ from the known $Q_{\text{ext}}({{\tilde{\nu }}})$. Thus, the task at hand is to solve an inverse scattering problem.

### Quality of reconstructions

The quality of the reconstructions is best assessed with the help of the coefficient of determination, $R^2$. Given the refractive-index data points, $\eta _k^\text{PMMA}$, $k=1,\ldots N_{\text{PMMA}}$^14^ and $\eta _k^\text{PS}$, $k=1,\ldots N_{\text{PS}}$^15^, and defining ${{\tilde{\eta }}}_k^\text{PMMA}$, $k=1,\ldots N_{\text{PMMA}}$ and ${{\tilde{\eta }}}_k^\text{PS}$, $k=1,\ldots N_{\text{PS}}$ as their reconstructions according to the Lorentzian method, we define the residuals, $e_k^\text{PMMA,PS}= \eta _k^\text{PMMA,PS}- {{\tilde{\eta }}}_k^\text{PMMA,PS}$ and the averages ${{\bar{\eta }}}_{\text{PMMA,PS}}= (1/N_{PMMA,PS})\sum _{k=1}^ {N_{\text{PMMA,PS}}} \eta _k^\text{PMMA,PS}$. We then define the residual sum of squares of the real and imaginary parts of the residuals according to $S_{\text{res}}^{(\mathrm PMMA,PS,r)}= \sum _{k=1}^{N_{\text{PMMA,PS}}} (\Re e_k^\text{PMMA,PS})^2$, $S_{\text{res}}^{(\mathrm PMMA,PS,i)}= \sum _{k=1}^{N_{\text{PMMA,PS}}}$ $(\Im e_k^\text{PMMA,PS})^2$, respectively. We also define the total sum of squares of the real and imaginary parts of the given and reconstructed refractive indexes according to $S_{\text{tot}}^\text{PMMA,PS,r}= \sum _{k=1}^{N_{\text{PMMA,PS}}} [\Re (\eta _k^\text{PMMA,PS}- {{\bar{\eta }}}_k^\text{PMMA,PS})]^2$ and $S_{\text{tot}}^\text{PMMA,PS,i}= \sum _{k=1}^{N_{\text{PMMA,PS}}} [\Im (\eta _k^\text{PMMA,PS}- {{\bar{\eta }}}_k^\text{PMMA,PS})]^2$. Then, the coefficient of determination is given by

7 $$\begin{aligned} \left[ R^\text{PMMA,PS,r,i} \right] ^2 = 1-\frac{S_{\text{res}}^\text{PMMA,PS,r,i}}{S_{\text{tot}}^\text{PMMA,PS,r,i}}. \end{aligned}$$

The $R^2$ value is a useful measure of the quality of reconstruction. For instance, an $R^2$ value of 0.5 indicates that 50% of the $Q_{\text{ext}}^{(\text{given})} ({{\tilde{\nu }}})$ data is explained by the reconstruction. As shown in “Results” section the $R^2$ values of our reconstructions are all above 0.7 and are above 0.9 if reconstructions are performed with high-quality $Q_{\text{ext}}^{(\text{given})} ({{\tilde{\nu }}})$ data as input (see “Reconstruction from numerical extinction data” section). Even for the case of a layered sphere, discussed in “Layered sphere: space-resolved refractive index” section, the $R^2$ value still exceeds 0.8 for both the core and shell reconstructions.

## Results

To demonstrate the power of the new algorithm, we extract the refractive index in three cases, where $Q_{\text{ext}}({{\tilde{\nu }}})$ is (i) numerically simulated (“Reconstruction from numerical extinction data” section), (ii) experimentally measured (real-life case, “Reconstruction from experimental extinction data” section), and (iii) numerically simulated for a layered sphere (“Layered sphere: space-resolved refractive index” section). While the algorithm straightforwardly scales to more complex space-dependent refractive indexes, case (iii) demonstrates explicitly, for a layered sphere, that the algorithm is capable of space-resolving the complex refractive index in a simple model case.

While these examples are important for testing validity and robustness of the Lorentzian method, IR spectroscopy on homogeneous and layered spheres may also have practical importance, for instance in mineralogy, or for characterizing micro- and nano-spheres, such as PMMA or PS spheres. PMMA spheres, in particular, are frequently used as model systems for biological cells^11,12,18–21^.

### Reconstruction from numerical extinction data

The purpose of this section is to show that the Lorentzian reconstruction method works near perfectly if provided with high-accuracy $Q_{\text{ext}}^{(\text{given})} ({{\tilde{\nu }}})$ data. To demonstrate the power of the Lorentzian method in this case, we computed, numerically, the extinction efficiency $Q_{\text{ext}}^{(\text{given})} ({{\tilde{\nu }}})$ for high-accuracy experimental refractive indexes for PMMA^14^ and PS^15^. For the numerical computation of $Q_{\text{ext}}^{(\text{given})} ({{\tilde{\nu }}})$ we used a standard Mie scattering code^16^. Given that $Q_{\text{ext}}^{(\text{given})} ({{\tilde{\nu }}})$ in this case exactly corresponds to the input refractive indexes, and would also correspond to very high-accuracy measurements of $Q_{\text{ext}}^{(\text{given})} ({{\tilde{\nu }}})$, we expect that the original refractive indexes that served as input for this numerical determination of $Q_{\text{ext}}^{(\text{given})} ({{\tilde{\nu }}})$ are reproduced with high accuracy. As shown in Fig. 2, this is indeed the case. Although starting with random initial conditions, as described in “Numerical simulations” section, far from the values that correspond to the functional behavior of the PMMA and PS refractive indexes, we obtain near perfect reconstructions of the refractive indexes of PMMA and PS, as demonstrated by the closeness of the red lines (reconstructions) to the blue lines (original PMMA and PS refractive indexes) in Fig. 2. As can be seen visually, the residuals of the reconstructions are very small. Quantitatively, the coefficients of determination $R^2$ (see “Quality of reconstructions” section) are 0.983, 0.979, 0.936, and 0.928 for Fig. 2a–d, respectively. Thus, the results of this section demonstrate that given high-quality $Q_{\text{ext}}^{(\text{given})} ({{\tilde{\nu }}})$ input data, the Lorentz reconstruction method performs almost perfectly. Neither a reference spectrum nor numerical Kramers-Kronig transformations were necessary for performing the reconstruction algorithm.

### Reconstruction from experimental extinction data

**Figure 3:**
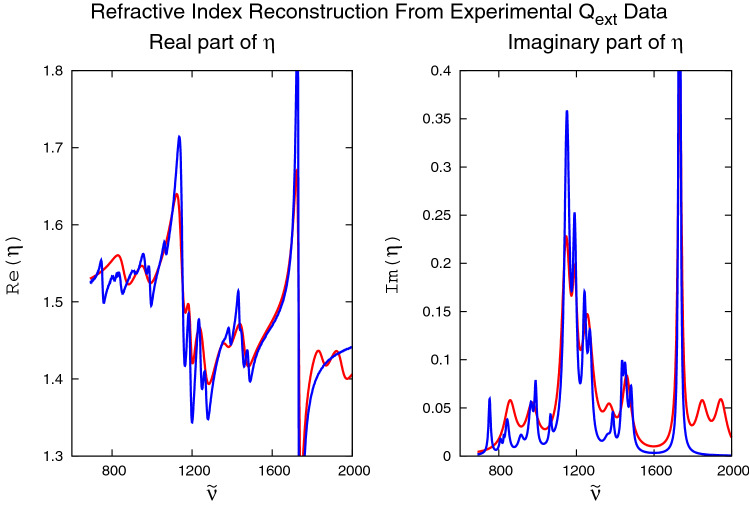
Real-life example of the reconstruction of real and imaginary parts of the complex refractive index $\eta ({{\tilde{\nu }}})$ of PMMA (red lines) for an experimental PMMA extinction spectrum of a PMMA sphere^19^ compared with the experimental refractive index^14^ of PMMA (blue lines). 18 Lorentzians were used for the reconstructions. Although the initial guess of the complex refractive index is random, the reconstructions (red lines) of real and imaginary parts of $\eta ({{\tilde{\nu }}})$ are close to their experimental values.

In the way of testing the Lorentz method on a real-life example, we reconstructed the complex PMMA refractive index from an experimental apparent absorbance spectrum^19^, simultaneously performing shift-, tilt-, curvature-, and scaling corrections of the the raw spectrum. This was accomplished by adding shift-, tilt-, curvature, and scaling parameters as additional fit parameters to the nonlinear optimization algorithm discussed in “Reconstruction of the complex refractive index” section. The result is shown in Fig. 3. Starting the Lorentzian reconstruction algorithm once more with random initial conditions, we obtained reconstructions (red lines) of the real and imaginary parts of the complex PMMA index of refraction in satisfactory agreement with the experimental data (blue lines). This time, the coefficients of determination $R^2$ (see “Quality of reconstructions” section) are 0.803 and 0.753 for Fig. 3a,b, respectively. While the reconstructions from experimental data are not as good as the reconstructions from numerical $Q_{\text{ext}}^{(\text{given})} ({{\tilde{\nu }}})$ data (which is to be expected, given the presence of experimental systematic and statistical errors that are not included in simple spectral shift-, tilt-, curvature-, and scaling deformations).

Figure 3 still confirms that the Lorentz reconstruction algorithm also works for experimental raw spectra that are neither scatter corrected, nor corrected for experimental systematic and random errors. Apparently, as shown in Fig. 3, the algorithm can handle both scatter corrections and experimental errors simultaneously without the need of a reference spectrum or a numerical Kramers–Kronig transformation. Additional wiggles in the reconstructions (red curves), not present in the experimental refractive-index data (blue lines) may be due to spurious oscillations induced by experimental errors in the experimental $Q_{\text{ext}}^{(\text{given})} ({{\tilde{\nu }}})$ data that served as input for the reconstructions. Similar arguments may apply to peaks in the experimental refractive-index data (blue lines) that are not captured by the reconstructions (red lines). In this case, it is possible that 18 Lorentzians may not be enough for more faithful reconstructions.

### Layered sphere: space-resolved refractive index

In “Reconstruction from numerical extinction data” and “Reconstruction from experimental extinction data” sections we focused on exploring the power of the Lorentzian method in the well-defined case of homogeneous spheres. We now ask the question whether the Lorentzian algorithm is powerful enough to obtain the space-resolved complex refractive index, i.e., space-resolved chemistry, via the imaginary part of the complex refractive index (absorption). To answer this question, we focus on a stratified sphere with two layers. The outer radius of the sphere is $10\,{\upmu }$m; the inner radius, i.e., the core radius, is $8 \,{\upmu }$m. We fill the core of the layered sphere with PMMA, the shell with PS, and computed $Q_{\text{ext}}^{(\text{given})} ({{\tilde{\nu }}})$ using a standard MATLAB code for stratified spheres^22^.

**Figure 4:**
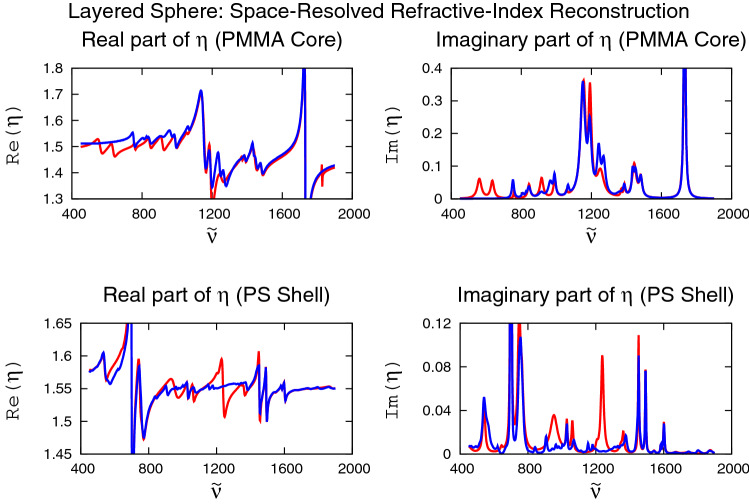
Reconstructions of real and imaginary parts of the complex refractive index $\eta ({{\tilde{\nu }}})$ (blue lines) for a layered sphere consisting of PMMA in the core and PS in the shell. 18 Lorentzians were used for the PMMA reconstructions and 22 Lorentzians were used for the PS reconstructions.

For the refractive indexes in the core and the shell we used experimental data^14,15^. Constructing $Q_{\text{ext}}^{(\text{given})} ({{\tilde{\nu }}})$ on the basis of this input, we obtained the reconstructions of the complex indexes of refraction in the core and in the shell as shown in Fig. 4. We see that, not only does the Lorentzian algorithm, based only on the extinction efficiency $Q_{\text{ext}}^{(\text{given})} ({{\tilde{\nu }}})$, correctly identify the two substances and their spatial locations (PMMA in the core and PS in the shell), the reconstructions themselves (red lines) are close to their actual values (blue lines), although, in certain wavenumber regions, we also see relatively large deviations. These deviations, however, are not large enough to prevent us from obtaining the correct ordering, i.e., space resolving the substances, i.e., PMMA in the core and PS in the shell. Quantitatively, the coefficients of determination $R^2$ (see “Quality of reconstructions” section) in the layered case are 0.924, 0.943, 0.867, and 0.862 for Fig. 4a–d, respectively. Thus, the $R^2$ values are not too far from their values for the high-accuracy case discussed in “Reconstruction from numerical extinction data” section. This is encouraging. The more so that, again, we started from completely unbiased, random initial conditions. This demonstrates that the Lorentzian algorithm is capable of revealing space-resolved chemistry based only on a single-element detector, i.e., only $Q_{\text{ext}}^{(\text{given})} ({{\tilde{\nu }}})$ as input.

We mention that we also used the Lorentzian method to discover the core radius, assumed unknown. In this case we added the core radius as an additional parameter to the Lorentzian method. We found that in this case the Lorentzian method is not only able to reconstruct the complex indexes of refraction, but simultaneously also determines the core radius. As a result, we find that we can reconstruct the complex refractive indexes, space-dependent, along with determining the core radius, which may represent the size of the nucleus of a biological cell.

## Discussion

In their natural state, biological cells have a 3D structure, which, combined with the fact that cells and tissues have sizes that are of the order of the IR wavelength, leads to large scattering contributions that need to be removed to obtain the complex refractive index, which only then can be interpreted as to its chemical information content. The new Lorentzian method, compact and powerful, accomplishes just that, and, as demonstrated in “Results” section, can even be combined with baseline-shift, tilt-, curvature-, and scaling corrections of raw spectra. The method is also suitable for extracting space-resolved chemistry as demonstrated in “Layered sphere: space-resolved refractive index” section in the case of a layered sphere.

Concerning stability and reliability of the Lorentzian method, we performed several checks. (a) We perturbed the simulated $Q_{\text{ext}}({{\tilde{\nu }}})$ spectrum with up to 10% of noise and found that reconstruction was still possible. (b) We always started our reconstructions with random inputs for peak widths and peak heights. (c) Apart from choosing equidistant peak locations, as described in “Numerical simulations” section, we also experimented with random initial conditions for the peak locations and in terms of $R^2$ values found comparably good results. Under all these perturbations and variations, we always find convergence to the correct complex refractive index. This demonstrates that the method is robust and does not require fine-tuning of input parameters.

With the advent of new IR sources, such as tunable quantum cascade lasers, IR spectroscopy has recently received a rejuvenating boost that offers a promising opportunity for the application of the Lorentzian method, as it works well with coherent illumination of samples. We mention that in the case of coherent IR sources, optical effects, such as interference and scattering are more important and more controlled as compared to thermal IR sources or (partially coherent) synchrotron sources. Thus, the Lorentzian method is ideally suited for obtaining the complex index of refraction in combination with coherent IR laser sources. We also mention that lasers are cheaper and more accessible than synchrotron sources, which makes the Lorentzian method ideally suited for laboratory applications.

A final, but important point concerns our choice of anti-symmetrized Lorentzian functions. Lorentzians are, in fact, not the only possible choice of basis functions for performing the reconstructions. Any complete set of functions may be chosen as long as they automatically satisfy the Kramers–Kronig relations. An example of an alternative basis set of anti-symmetrized basis functions is, e.g., the set of anti-symmetrized Gaussian functions^17^.

## Conclusion

In this paper we presented a new reconstruction algorithm that is capable of extracting space- and wavenumber-resolved complex refractive indexes from the extinction efficiency $Q_{\text{ext}}^{(\text{given})} ({{\tilde{\nu }}})$ of strongly scattering biological and inanimate samples. We illustrated the capabilities of the algorithm with the help of three examples, i.e., two model spheres that are homogeneously filled with PMMA and PS, an experimentally measured (real-life) PMMA sphere, and a layered sphere with PMMA in its core and PS in its shell. In all cases satisfactory reconstructions of the complex refractive index were obtained. The most appealing feature of this new algorithm is that it requires neither a reference spectrum nor a numerical Kramers–Kronig transformation. Due to its clear formulation, its straightforward implementation, and its convenient features, we are convinced that this method may find practical applications in biophysics, medical diagnostics, medical pathology, and single-cell spectroscopy.

## Data Availability

The datasets used and/or analysed during the current study are available from the corresponding author on reasonable request.

## References

[1] Barnes RB, Bonner LG (1936). The early history and the methods of infrared spectroscopy. Am. J. Phys..

[2] Bhargava R (2012). Infrared spectroscopic imaging: The next generation. Appl. Spectrosc..

[3] Mohlenhoff B, Romeo M, Diem M, Wood BR (2005). Mie-type scattering and non-Beer–Lambert absorption behavior of human cells in infrared microspectroscopy. Biophys. J ..

[4] Ferrari M, Quaresima V (2012). A brief review on the history of human functional near-infrared spectroscopy (fNIRS) development and fields of application. Neuroimage.

[5] Lukacs R, Blümel R, Zimmerman B, Bağcıoğlu M, Kohler A (2015). Recovery of absorbance spectra of micrometer-sized biological and inanimate particles. Analyst.

[6] Jamin N (1998). Highly resolved chemical imaging of living cells by using synchrotron infrared microspectrometry. Proc. Natl. Acad. Sci. U.S.A..

[7] Haas J, Mizaikoff B (2016). Advances in mid-infrared spectroscopy for chemical analysis. Annu. Rev. Anal. Chem..

[8] Solheim JH (2019). An open-source code for Mie extinction extended multiplicative signal correction for infrared microscopy spectra of cells and tissues. J. Biophotonics.

[9] Bassan P (2010). Resonant Mie scattering (RMieS) correction of infrared spectra from highly scattering biological samples. Analyst.

[10] Kohler A (2008). Estimating and correcting Mie scattering in synchrotron-based microscopic Fourier transform infrared spectra by extended multiplicative signal correction. Appl. Spectrosc..

[11] Van Dijk T, Mayerich D, Carney PS, Bhargava R (2013). Recovery of absorption spectra from Fourier transform infrared (FT-IR) microspectroscopic measurements of intact spheres. Appl. Spectrosc..

[12] Rasskazov IL, Spegazzini N, Carney PS, Bhargava R (2017). Dielectric sphere clusters as a model to understand infrared spectroscopic imaging data recorded from complex samples. Anal. Chem..

[13] Hulst HC (1981). Light Scattering by Small Particles.

[14] Tsuda S, Yamaguchi S, Kanamori Y, Yugami H (2018). Spectral and angular shaping of infrared radiation in a polymer resonator with molecular vibrational modes. Opt. Express.

[15] Myers TL (2018). Accurate measurement of the optical constants $$n$$ and $$k$$ for a series of 57 inorganic and organic liquids for optical modeling and detection. Appl. Spectrosc..

[16] Mätzler, C. *MATLAB Functions for Mie Scattering and Absorption, Version 2, Research Report No. 2002-11. Tech. Rep*. (Institut für Angewandte Physik, Universität Bern, 2002).

[17] Keefe CD (2001). Curvefitting imaginary components of optical properties: Restrictions on the lineshape due to causality. J. Mol. Spectrosc..

[18] Bassan P (2009). Resonant Mie scattering in infrared spectroscopy of biological materials-understanding the ‘dispersion artefact’. Analyst.

[19] Blümel R, Bağcioğlu M, Lukacs R, Kohler A (2016). Infrared refractive index dispersion of polymethyl methacrylate spheres from Mie ripples in Fourier-transform infrared microscopy extinction spectra. J. Opt. Soc. Am. A.

[20] Matamoros-Ambrocio M, Sánchez-Mora E, Gómez-Barojas E, Luna-López JA (2021). Synthesis and study of the optical properties of PMMA microspheres and opals. Polymers.

[21] Brandsrud MA, Blümel R, Solheim JH, Kohler A (2021). The effect of deformation of absorbing scatterers on Mie-type signatures in infrared microspectroscopy. Sci. Rep..

[22] Gibson, W. *Scattered Field of a Conducting and Stratified Spheres. Tech. Rep, MATLAB Central File Exchange*. https://www.mathworks.com/matlabcentral/fileexchange/20430-scattered-field-of-a-conducting-and-stratified-spheres (2022).

[23] Griffiths DJ (1999). Introduction to Electrodynamics.

[24] Mayerhöfer TG, Popp J (2019). Quantitative evaluation of infrared absorbance spectra - Lorentz profile versus Lorentz oscillator. ChemPhysChem.

[25] Mayerhöfer TG, Ivanovski V, Popp J (2022). Infrared refraction spectroscopy—Kramers–Kronig analysis revisited. Spectrochim. Acta A Mol. Biomol. Spectrosc..

